# Simple models to include influenza vaccination history when evaluating the effect of influenza vaccination

**DOI:** 10.2807/1560-7917.ES.2021.26.32.2001099

**Published:** 2021-08-12

**Authors:** Iván Martínez-Baz, Ana Navascués, Itziar Casado, Aitziber Aguinaga, Carmen Ezpeleta, Jesús Castilla

**Affiliations:** 1 Instituto de Salud Pública de Navarra, Pamplona, Spain; 2 CIBER Epidemiología y Salud Pública (CIBERESP), Pamplona, Spain; 3 Instituto de Investigación Sanitaria de Navarra (IdiSNA), Pamplona, Spain; 4 Servicio de Microbiología Clínica, Complejo Hospitalario de Navarra, Pamplona, Spain

**Keywords:** influenza vaccine effectiveness, influenza vaccination effect, prior vaccination, repeated vaccination, modifying effect, influenza vaccine

## Abstract

**Background:**

Most reports of influenza vaccine effectiveness consider current-season vaccination only.

**Aim:**

We evaluated a method to estimate the effect of influenza vaccinations (EIV) considering vaccination history.

**Methods:**

We used a test-negative design with well-documented vaccination history to evaluate the average EIV over eight influenza seasons (2011/12–2018/19; n = 10,356). Modifying effect was considered as difference in effects of vaccination in current and previous seasons and current-season vaccination only. We also explored differences between current-season estimates excluding from the reference category people vaccinated in any of the five previous seasons and estimates without this exclusion or only for one or three previous seasons.

**Results:**

The EIV was 50%, 45% and 38% in people vaccinated in the current season who had previously received none, one to two and three to five doses, respectively, and it was 30% and 43% for one to two and three to five prior doses only. Vaccination in at least three previous seasons reduced the effect of current-season vaccination by 12 percentage points overall, 31 among outpatients, 22 in 9–65 year-olds, and 23 against influenza B. Including people vaccinated in previous seasons only in the unvaccinated category underestimated EIV by 9 percentage points on average (31% vs 40%). Estimates considering vaccination of three or five previous seasons were similar.

**Conclusions:**

Vaccine effectiveness studies should consider influenza vaccination in previous seasons, as it can retain effect and is often an effect modifier. Vaccination status in three categories (current season, previous seasons only, unvaccinated) reflects the whole EIV.

## Introduction

The World Health Organization recommends annual influenza vaccination in the target population [1]. People vaccinated against influenza in one season are more likely to be vaccinated in the next one [2]; therefore those in the target population frequently accumulate influenza vaccines over the years. 

Influenza vaccines received in previous seasons may retain a notable protective effect [3-5]. In contrast with that, frequent prior vaccination has been related with a reduced effect (i.e. effect modifier) of the current-season vaccine in some studies [6-8]. Since influenza vaccination history is associated with a higher probability of current season vaccination and a lower risk of influenza, it is also a potential confounding factor of the current-season influenza vaccine effectiveness (IVE) [9-11].

IVE studies usually aim at assessing how each seasonal vaccine composition has worked against the circulating influenza viruses in real-life conditions, when frequently repeated vaccination is the most common scenario. However, most of these studies consider only the current-season vaccination [12], and those that consider previous vaccines usually do so as a secondary analysis [3,13].

The estimates from IVE studies are also used to inform population and health professionals about how much influenza vaccination reduces the risk of influenza outcomes; they are also used to estimate the impact of influenza vaccination in the population. For these objectives, it seems more adequate to estimate the effect of influenza vaccinations (EIV) considering both the current-season vaccination and the vaccination history. The inclusion of people only vaccinated in prior seasons in the reference category of unvaccinated may underestimate the EIV.

Influenza epidemics and IVE show important variability among seasons because vaccine composition and circulating virus strains change. The pooled analysis of multiple seasons provides average patterns that can guide public health recommendations.

Given the annual recommendation for influenza vaccination, this study’s aim was to evaluate how vaccination history affects the estimates of current-season IVE and to determine the simplest method to obtain valid estimates of the EIV for people according to their influenza vaccination status in the current and previous seasons.

## Methods

### Design and setting

This methodological proposal is illustrated with data from patients attended in primary healthcare centres and hospitals in the region of Navarre, Spain, during influenza seasons 2011/12 to 2018/19. Annual IVE studies have been conducted since 2009 using the test-negative case–control study design [14], nested in the cohort of the population covered by the Regional Health Service [8,15-21]. This Health Service provides healthcare, free at point of service, to 97% of the population. The trivalent inactivated influenza vaccine was recommended annually and offered free of charge to people 60 years or older (although the uptake was higher from age 65 years upwards) and to those with risk factors or major chronic conditions. Other people could also be vaccinated if they paid for the vaccine.

### Case definition and information sources

Influenza surveillance was based on automatic reporting of cases of medically attended influenza-like illness (ILI) from all primary healthcare centres and hospitals [18]. ILI was defined as the sudden onset of any general symptom (fever, malaise, headache or myalgia) in addition to any respiratory symptom (cough, sore throat or dyspnoea). A sentinel network composed of a representative sample of primary healthcare physicians took double swabs, nasopharyngeal and pharyngeal, after obtaining verbal informed consent, from all their patients diagnosed with ILI whose symptoms had begun fewer than 5 days before the patient consultation. The protocol for influenza cases in hospitals establishes early detection and nasopharyngeal and pharyngeal swabbing at admission of all hospitalised patients with ILI. Swabs were tested for influenza viruses by reverse transcription PCR (RT-PCR).

For the present study, we used the test-negative case–control design over eight influenza seasons, from 2011/12 to 2018/19. We included only patients with continued residence in the region during the previous 5 years. Children younger than 9 years, healthcare workers and nursing home residents were excluded. Cases were ILI patients of primary healthcare centres or hospitals, who were confirmed positive for influenza virus by RT-PCR, and controls were similar patients who tested negative for any influenza virus. The influenza vaccination status in the current season and vaccination history were obtained from the regional vaccination register, and only registered doses were considered [2]. Patients who had received an influenza vaccine fewer than 14 days before symptom onset were excluded.

### Statistical analysis

Characteristics of study participants by confirmed influenza status and current-season influenza vaccination were compared by chi-squared test. Logistic regression was used to calculate the odds ratios (OR) with their 95% confidence intervals (CI). All models were adjusted for age group (9–44, 45–64, 65–84 and ≥ 85 years), major chronic conditions, calendar month and season of sample collection, and for healthcare setting (primary healthcare or hospital). The IVE and EIV were estimated as (1 − adjusted OR) × 100%.

We evaluated different categorisations of the vaccination status. We considered the reference values to be the EIV estimates obtained from the ‘full model’ with the vaccination status in six categories: current-season vaccination and three to five prior doses, current-season vaccination and one to two prior doses, current-season vaccination and no prior doses, three to five prior doses and no current-season vaccination, one to two prior doses and no current-season vaccination, and unvaccinated in the current and five previous seasons as the reference category [6-8].

The modifying effect of previous seasons’ vaccines on current-season IVE was evaluated in the full model by the absolute difference in effectiveness of each vaccination status in comparison with the category of current-season vaccination and no prior doses as reference. When this difference was statistically significant (p < 0.05), modifying effect of vaccination history on the current-season vaccine effectiveness was concluded, and the final results were those from combining vaccination categories of the current and previous seasons. Interaction terms between current-season vaccination and prior doses received were tested and these results are presented in the Supplement.

As the full model requires high sample size, estimates from alternate models were compared with those from the full model to determine the analysis with the fewest requirements of vaccination history data and successful management of the remaining and modifying effects of prior vaccines. We also tested models combining vaccination status in the current and either one or three immediately preceding seasons, as well as the model considering only the current-season influenza vaccination (only-current-season model). When the three estimates of the current-season IVE provided by the full model were not statistically different, the analysis was summarised in a ‘summarised model’ with only three categories: current-season vaccination regardless of prior doses, no current-season vaccination but any prior doses, and neither current-season vaccination nor any prior doses as the reference category [22].

The inclusion in the reference category of individuals vaccinated only in previous seasons may bias the EIV estimates. This bias was evaluated as the absolute difference in estimates as ∆IVE = IVE_0_ – IVE_1_; where IVE_1_ was the current-season EIV estimate in the summarised model considering vaccination in the five preceding seasons, and IVE_0_ was the current-season IVE estimate in either the only-current-season model or the summarised models considering vaccination in either one or three previous seasons. A ∆IVE of five or more percentage points was considered as a bias and of 10 or more as relevant bias [23,24].

We tested the proposal in separated analyses by healthcare setting, age group, virus (sub-)type and influenza season. Seasons with minimal circulation of a given (sub-)type were excluded from the pooled analysis for that outcome. In sensitivity analyses, we included the diagnoses of ILI in the immediately preceding season as a covariable.

## Ethical statement

The Navarra Ethical Committee for Medical Research approved the study protocol (Pyto 85/11, Pyto 2015/95 and Pyto 2017/88).

## Results

### Characteristics of participants

During the eight influenza seasons studied, 10,356 swabbed patients were enrolled: 4,412 patients attended in primary healthcare and 5,944 were hospitalised patients, of whom respectively 2,872 (65%) and 2,069 (35%) were confirmed for influenza virus infection.

Table 1 describes the participants’ characteristics by influenza case status and current vaccination status. Among the total number of patients, 46% were 65 years or older and 38% had received the influenza vaccine in the current season. Current season vaccination was less frequent in cases than in controls (29% vs 44%; p < 0.001). Influenza A(H3N2) accounted for almost half of the cases (47%), while influenza A(H1N1) and B were equally frequent (26% and 27%, respectively) (Supplementary Table S1). 

**Table 1:** Participant profile, by influenza case status and current vaccination status, Navarre, Spain, pooled analysis of 2011/12–2018/19 seasons (n = 10,356)

	Laboratory-confirmed cases		Negative controls		p value	Vaccinated in current season		Unvaccinated in current season		p value
	n	%	n	%		n	%	n	%	
Total	4,941	100	5,415	100	NA	3,952	100	6,404	100	NA
Age groups (years)										
9–44	1,798	36	1,279	24	< 0.001	265	7	2,812	44	< 0.001
45–64	1,328	27	1,194	22		516	13	2,006	31	
65–84	1,342	27	2,076	38		2,189	55	1,229	19	
≥ 85	473	10	866	16		982	25	357	6	
Sex										
Male	2,510	51	2,837	52	0.105	2,089	53	3,258	51	0.050
Female	2,431	49	2,578	48		1,863	47	3,146	49	
Major chronic conditions										
No	2,437	49	1,794	33	< 0.001	636	16	3,595	56	< 0.001
Yes	2,504	51	3,621	67		3,316	84	2,809	44	
Healthcare setting										
Primary care	2,872	58	1,540	28	< 0.001	693	18	3,719	58	< 0.001
Hospital	2,069	42	3,875	72		3,259	82	2,685	42	
Influenza-like illness diagnosis in the immediately preceding season										
No	4,806	97	5,248	97	0.288	3,887	98	6,167	96	< 0.001
Yes	135	3	167	3		65	2	237	4	
Vaccination in the current season										
No	3,500	71	2,904	54	< 0.001	NA				
Yes	1,441	29	2,511	46						
Vaccination in the previous season										
No	3,561	72	2,974	55	< 0.001	627	16	5,908	92	< 0.001
Yes	1,380	28	2,441	45		3,325	84	496	8	
Vaccines in the five previous seasons										
0	3,191	65	2,410	45	< 0.001	283	7	5,318	83	< 0.001
1–2	402	8	625	12		454	11	573	9	
3–5	1,348	27	2,380	44		3,215	81	513	8	
Influenza season										
2011/12	351	7	235	4	< 0.001	96	2	490	8	< 0.001
2012/13	315	6	258	5		92	2	481	8	
2013/14	527	11	476	9		331	8	672	10	
2014/15	573	12	482	9		325	8	730	11	
2015/16	734	15	703	13		457	12	980	15	
2016/17	662	13	878	16		711	18	829	13	
2017/18	1,044	21	1,167	22		1,029	26	1,182	18	
2018/19	735	15	1,216	22		911	23	1,040	16	

NA: not applicable.

Influenza vaccination in previous seasons met the conditions for being a potential confounding factor in the analysis of IVE, since it was more frequent among controls than in cases (55% vs 35%; p < 0.001) and among those patients vaccinated in the current season than in the rest (93% vs 17%; p < 0.001). 

### Effect of vaccination in the current and previous seasons

Compared with persons unvaccinated in the current and the five previous seasons, those vaccinated in the current season had an average protective effect of 50% (95% CI: 35–62), 45% (95% CI: 31–56) and 38% (95% CI: 30–45) if they had received none, one to two and three to five prior doses of vaccine, respectively. Persons unvaccinated in the current season also experienced a protective effect of 30% (95% CI: 16–42) and 43% (95% CI: 29–53) if they had received one to two or three to five prior doses, respectively. The EIV estimates from the full models considering combination of vaccination status in the current and previous seasons did not show relevant differences when three or five previous seasons were considered, but the differences increased when only one previous season was considered (Table 2).

**Table 2:** Effect of influenza vaccination in the current and previous seasons for all patients and by healthcare setting, Navarre, Spain, pooled analysis of the 2011/12–2018/19 seasons (n = 10,356)

	**All patients**				**Primary healthcare patients**				**Hospital patients**			
	**Cases**	**Controls**	**Vaccination effect**		**Cases**	**Controls**	**Vaccination effect**		**Cases**	**Controls**	**Vaccination effect**	
			**%**	**95% CI^ a^**			**%**	**95% CI^ a^**			**%**	**95% CI^ a^**
Only-current-season model												
Unvaccinated	3,500	2,904	0 (ref)		2,474	1,245	0 (ref)		1,026	1,659	0 (ref)	
Vaccinated	1,441	2,511	31	23–38	398	295	35	21–47	1,043	2,216	29	19–37
Summarised model with one previous season												
Never vaccinated	3,333	2,575	0 (ref)		2,433	1,210	0 (ref)		900	1,365	0 (ref)	
Prior and no current	167	329	41	27–52	41	35	42	7–64	126	294	40	24–53
Current regardless prior	1,441	2,511	36	29–43	398	295	37	23–48	1,043	2,216	36	27–44
Full model with one previous season												
Never vaccinated	3,333	2,575	0 (ref)		2,433	1,210	0 (ref)		900	1,365	0 (ref)	
Prior and no current	167	329	41	27–52	41	35	41	6–64	126	294	40	24–53
Current and no prior	228	399	40	28–51	85	84	53	35–66	143	315	33	16–47
Current and prior	1,213	2,112	35	27–42	313	211	28	10–43^b^	900	1,901	36	27–44
Summarised model with three previous seasons												
Never vaccinated	3,179	2,351	0 (ref)		2,385	1,168	0 (ref)		794	1,183	0 (ref)	
Any prior and no current	321	553	37	26–47	89	77	46	25–61	232	476	35	21–46
Current regardless prior	1,441	2,511	39	32–45	398	295	39	25–50	1,043	2,216	38	29–46
Full model with three previous seasons												
Never vaccinated	3,179	2,351	0 (ref)		2,385	1,168	0 (ref)		794	1,183	0 (ref)	
1–2 prior and no current	235	362	30	16–42	79	61	40	14–58	156	301	27	9–42
3 prior and no current	86	191	49	33–62	10	16	69	29–86	76	175	47	28–61
Current and no prior	118	215	50	36–61	58	65	59	41–72	60	150	41	18–57
1–2 prior and current	352	631	40	29–49	108	82	38	14–55	244	549	41	28–51
3 prior and current	971	1,665	36	28–44	232	148	27	4–44^b^	739	1,517	37	27–46
Summarised model with five previous seasons												
Never vaccinated	3,089	2,229	0 (ref)		2,345	1,135	0 (ref)		744	1,094	0 (ref)	
Any prior and no current	411	675	37	26–46	129	110	46	28–59	282	565	33	20–4
Current regardless prior	1,441	2,511	40	33–47	398	295	40	27–51	1,043	2,216	39	30–47
Full model with five previous seasons												
Never vaccinated	3,089	2,229	0 (ref)		2,345	1,135	0 (ref)		744	1,094	0 (ref)	
1–2 prior and no current	237	336	30	16–42	103	79	37	14–54	134	257	25	5–41
3–5 prior and no current	174	339	43	29–53	26	31	63	36–79	148	308	39	23–52
Current and no prior	102	181	50	35–62	53	59	59	39–72	49	122	40	14–58
1–2 prior and current	165	289	45	31–56	67	58	47	23–64	98	231	42	24–56
3–5 prior and current	1,174	2,041	38	30–45	278	178	28	7–44^b^	896	1,863	39	29–47

CI: confidence interval; ref: reference.

^a^ Vaccination effect adjusted by age groups (9–44, 45–64, 65–84 and ≥ 85 years), major chronic conditions and month and season of sample collection.

^b^ p value < 0.05 for comparison with the category of current season vaccination and no prior doses.

In the sensitivity analysis including the ILI diagnosis during the immediately previous season, the EIV estimates were not subject to relevant changes (Supplementary Table S2).

### Modifying effect of prior doses on the current-season influenza vaccine effectiveness

**Figure 1:**
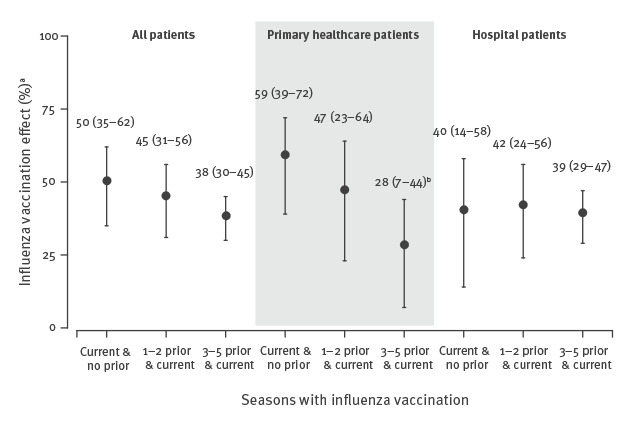
Evaluation of the modifying effect of vaccination history on the effect of influenza vaccination in the current season for all patients and by healthcare setting, Navarre, Spain, pooled analysis of the 2011/12–2018/19 influenza seasons (n = 10,356)

^a^ Influenza vaccination effect and 95% confidence interval (in brackets), adjusted by age groups (9–44, 45–64, 65–84 and ≥ 85 years), major chronic conditions, healthcare setting (primary healthcare and hospital) and month and season of sample collection.

^b^ p value < 0.05 for comparison with the category of current season vaccination and no prior doses.

**Figure 2:**
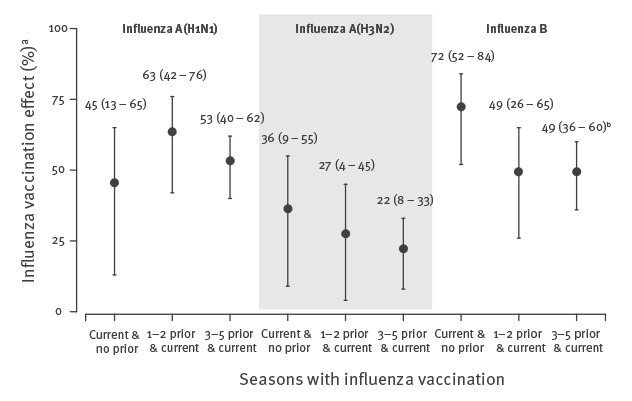
Evaluation of the modifying effect of vaccination history on the effect of influenza vaccination in the current season by influenza virus (sub)type, Navarre, Spain, pooled analysis of the 2011/12–2018/19 influenza seasons (n = 10,356)

^a^ Influenza vaccination effect and 95% confidence interval (in brackets), adjusted by age groups (9–44, 45–64, 65–84 and ≥ 85 years), major chronic conditions, healthcare setting (primary healthcare and hospital) and month and season of sample collection. Influenza A(H1N1) analysis includes 2012/13, 2013/14, 2015/16, 2017/18 and 2018/19 seasons. Influenza A(H3N2) analysis includes 2011/12, 2013/14, 2014/15, 2015/16, 2016/17, 2017/18 and 2018/19 seasons. Influenza B analysis includes 2011/12, 2012/13, 2014/15, 2015/16 and 2017/18 seasons (excluding seasons with minimal circulation of a given (sub-)type from the pooled analysis for that outcome).

^b^ p value < 0.05 for comparison with the category of current season vaccination and no prior doses.

In the overall analysis, the absolute difference of IVE of patients vaccinated in the current and in three or more previous seasons minus IVE of patients vaccinated in the current season and unvaccinated in the five previous seasons was −12 percentage points (38% vs 50%; p = 0.118). This difference reached −31 percentage points in primary healthcare patients (28% vs 59%; p = 0.014), −22 points among patients aged 9–64 years (36% vs 58%; p = 0.046), and −23 points in the analysis of influenza B cases (49% vs 72%; p = 0.037) (Figures 1 and 2).

Therefore, all these analyses should maintain separate estimates by vaccination history. Such negative interference was not observed in hospitalised patients, in patients 65 years or older, or in analyses of influenza A(H1N1) and A(H3N2); therefore, the vaccination categories might be simplified in those analyses. The full models considering the vaccination history in three or five previous seasons provided similar results, supporting the use of the first option (Table 2 and Supplementary Tables S3–S8).

In separate analyses by influenza season, the absolute difference in IVE ranged from −3 to +14 percentage points, but the sample sizes were limited and none of these differences had p < 0.05 (Supplementary Table S9).

### Estimating the bias from prior vaccinations in the reference category

We reduced the number of vaccination categories by combining all individuals vaccinated in the current season regardless of their vaccination history, and these were compared with subjects unvaccinated in the current and five previous seasons. Compared with this summarised model, the only-current-season model underestimated the EIV by an average of 9 percentage points (IVE = 31% vs EIV = 40%; absolute difference −9%), as did the summarised model considering vaccination in only one previous season (−4%) (Table 3). The only-current-season model was affected by a bias of at least 5 percentage points in the analysis of primary healthcare patients (−5%), hospitalised patients (−10%), patients 65 years and older (−11%), and in the analyses of influenza A(H1N1) (−6%), A(H3N2) (−10%) and influenza B (−9%). The bias also reached at least 5 percentage points in the summarised model with vaccination history in only one previous season in the analyses of influenza B (−6%; Table 3). No bias was observed in the summarised models including vaccination history in three previous seasons. The bias in the only-current-season model ranged from −1 to −19 percentage points among influenza seasons, and was at least 5 percentage points in five of the eight seasons studied (Supplementary Table S10).

**Table 3:** Comparison of the effect estimates of influenza vaccination from different models with the estimate that compares people vaccinated in the current season with those unvaccinated in the current and five previous seasons, Navarre, Spain, pooled analysis of 2011/12–2018/19 influenza seasons (n = 10,356)

	**Vaccination effect**		**Absolute difference of vaccination effect in %**
	**%**	**95% CI^ a^**	
All patients			
Summarised model with five previous seasons	40	33–47	Reference
Summarised model with three previous seasons	39	32–45	−1
Summarised model with one previous season	36	29–43	−4
Only-current-season model	31	23–38	−9^b^
Primary healthcare patients^c^			
Summarised model with five previous seasons	40	27–51	Reference
Summarised model with three previous seasons	39	25–50	−1
Summarised model with one previous season	37	23–48	−3
Only-current-season model	35	21–47	−5^b^
Hospitalised patients			
Summarised model with five previous seasons	39	30–47	Reference
Summarised model with three previous seasons	38	29–46	−1
Summarised model with one previous season	36	27–44	−3
Only-current-season model	29	19–37	−10^b,d^
Age 9–64 years^c^			
Summarised model with five previous seasons	45	35–54	Reference
Summarised model with three previous seasons	46	34–53	1
Summarised model with one previous season	43	32–52	−2
Only-current-season model	42	31–51	−3
Age ≥ 65 years			
Summarised model with five previous seasons	35	24–44	Reference
Summarised model with three previous seasons	34	23–44	−1
Summarised model with one previous season	32	21–41	−3
Only-current-season model	24	13–33	−11^b,d^
Influenza A(H1N1)			
Summarised model with five previous seasons	53	42–61	Reference
Summarised model with three previous seasons	50	39–60	−3
Summarised model with one previous season	51	40–59	−2
Only-current-season model	47	36–56	−6^b^
Influenza A(H3N2)			
Summarised model with five previous seasons	24	11–34	Reference
Summarised model with three previous seasons	24	13–35	0
Summarised model with one previous season	21	9–32	−3
Only-current-season model	14	2–25	−10^b,d^
Influenza B^c^			
Summarised model with five previous seasons	53	42–61	Reference
Summarised model with three previous seasons	50	39–59	−3
Summarised model with one previous season	47	35–56	−6^b^
Only-current-season model	44	32–53	−9^b^

CI: confidence interval.

^a^ Vaccination effect adjusted by age groups (9–44, 45–64, 65–84 and ≥ 85 years), major chronic conditions, healthcare setting (primary healthcare and hospital) and month and season of sample collection.

^b^ Absolute difference of vaccine effect of at least ± 5% was considered as a bias.

^c^ The results of this model should be considered with caution since there was a negative interference between vaccination in the current and previous seasons.

^d^ Absolute difference of vaccine effect of at least ± 10% was considered as a relevant bias.

### Selection of the simplest valid model to estimate the effect of influenza vaccinations

The EIV estimates in primary healthcare patients, in patients younger than 65 years and against influenza B were modified by the vaccination history; therefore, the analysis recommended in these situations is the full model which considers the vaccination history in at least three previous seasons. EIV estimates in the overall analysis and the specific analyses of hospitalised patients, of patients 65 years and older and against influenza A(H1N1) and A(H3N2) were not affected by a modifying effect, but were biased (± 5% or more) when at least one previous season vaccination was not considered; therefore the summarised model considering one prior dose may be sufficient (Table 4). When only relevant bias was considered (± 10% or more), the only-current-season model may be sufficient in the overall and in the specific analysis of influenza A(H1N1), but the summarised model would be the recommended analysis for hospitalised patients, patients 65 years and older and influenza A(H3N2).

**Table 4:** Simplest recommended model for estimating the influenza vaccination effect according to the modifying effect and bias from influenza vaccination history

	Modifying effect	Bias ± 5% or more	Relevant bias ± 10% or more	Simplest model recommended
**All patients**	**No**	**Yes**	**No**	**Summarised model with one previous season^a,b^**
Primary healthcare patients	Yes	Yes	No	Full model with three previous seasons^c^
Hospitalised patients	No	Yes	Yes	Summarised model with one previous season^a^
Age 9–64 years	Yes	No	No	Full model with three previous seasons^c^
Age ≥ 65 years	No	Yes	Yes	Summarised model with one previous season^a^
Influenza A(H1N1)	No	Yes	No	Summarised model with one previous season^a,b^
Influenza A(H3N2)	No	Yes	Yes	Summarised model with one previous season^a^
Influenza B	Yes	Yes	No	Full model with three previous seasons^c^

^a^ Summarised model categories: current-season vaccination regardless of prior doses, no current-season vaccination but any prior doses and no current-season vaccination or prior doses as reference category.

^b^ If only relevant bias is considered (± 10% or more), the only-current-season model would be sufficient.

^c^ Full model categories: current-season vaccination and three prior doses, current-season vaccination and one to two prior doses, current-season vaccination and no prior doses, no current-season vaccination and three prior doses, no current-season vaccination and one to two prior doses, and no current-season vaccination and no prior doses as reference category.

## Discussion

Influenza vaccination in previous seasons retained a considerable protective effect and in some cases modified the effect of current-season vaccination. Most analyses that did not consider vaccination in the previous seasons underestimated the EIV. Thus, evaluations of EIV should incorporate previous vaccination history.

In the analyses of patients in primary healthcare, of persons younger than 65 years, and of influenza B, people who were first vaccinated in the current season had an IVE more than 20 percentage points higher than those who had received the vaccine in the current season and in three or more previous seasons. A new dose of influenza vaccine in a person vaccinated in previous seasons could act by adding its effect to the remaining effect, or by maintaining the greater effect of both. On average over eight influenza seasons, the results observed do not support the sum of effects; some were consistent with maintaining the greater effect; and in various situations, persons with repeated vaccinations had lower protection than those vaccinated for the first time, suggesting there is negative interference (modification of the effect) between influenza vaccines, as described in other studies [7,19,25-29]. In these situations, the final analysis should estimate the effect separately for different combinations of vaccination in the current and previous seasons (full model).

Regardless of the presence of an effect modification, the analyses that did not remove from the reference category individuals vaccinated in only previous seasons underestimated by an average of 9 percentage points (IVE = 31% vs EIV = 40%) the preventive effect enjoyed by people vaccinated in the current season. This was a relevant bias (± 10% or more) in hospitalised patients, in those 65 years and older, and in cases of A(H3N2) influenza. Incorporating vaccination in the immediately previous season into the model largely reduced the bias, and the estimate was even improved by incorporating information from the three previous seasons. Although vaccines received 4–5 years ago may retain some residual effect [5], they did not substantially modify the current-season vaccine effectiveness [10].

Once the existence of an effect modification had been ruled out, the summarised model was a good option for evaluation of EIV, since it controlled the bias, estimated the protective effect in persons vaccinated in the current season, provided information about the remaining effect of previous vaccinations and did not cause excessive fragmentation of the results. While the analysis that considers only vaccination in the current season attempted unsuccessfully to isolate the effect of this vaccine, the summarised model assumed the reality of repeated vaccination and estimated the average protective effect in vaccinated individuals.

Although this study aimed to manage the bias and modifying effect in IVE estimates that inform population and health professionals about the risk reduction of influenza outcomes, similar modifying effect and bias may also affect studies that evaluate the effectiveness of a specific vaccine composition against the circulating influenza virus. Most studies published to date have not taken into account the history of previous vaccination [12], which suggests that the actual protection in vaccinated persons would have been higher than reported in the literature. Since the level of IVE is often low or moderate [12], it is essential to correct for this bias to strengthen the confidence of health professionals and the general population in vaccination, and thus improve vaccination coverage [30]. For many studies, it may be challenging to obtain reliable data on prior vaccination.

Natural immunity due to prior influenza exposure could introduce a bias in IVE and EIV studies [31]. The sensitivity analysis including the diagnoses of ILI during the immediately preceding season showed that the IVE estimates did not suffer relevant changes; therefore, this adjustment in the analysis does not seem necessary, as had been reported [10,18].

Among the strengths of this study is that it analysed an average of eight seasons with circulation of different (sub-)types of influenza virus. Including general practice and hospital settings provides complementary views of the EIV in the same population [20]. Cases were compared with controls recruited in the same healthcare settings before either patient or physician knew the laboratory result, a fact that reduced selection bias [14,32]. The vaccination history was obtained from the regional vaccination registry [2], and the study was limited to the population with stable residence in the region to avoid biases due to vaccination information [33]. The full model has been used in other studies and allows analysis of the effect modification and control for bias due to vaccination in previous seasons [6-8].

This study may also be subject to limitations. It was carried out in a single place where vaccination is indicated in persons 60 years and older and in those with risk factors, and only using the inactivated trivalent vaccine. Consequently, care must be taken when generalising the EIV results to other places with different indications for vaccination, different vaccination coverage, or where other types of vaccines are used. This study included individuals with different chances for repeated vaccination based on their age and influenza vaccination recommendation; therefore, all analyses were adjusted by age, comorbidities and season to control for potential confounding. The test-negative design in inpatient settings may be affected by bias, but the adjustment for cardiorespiratory conditions, as we have done, prevents this bias [34]. As the statistical power in the analysis of a single season was reduced, caution should be exercised when explaining its results. The results should be understood as an average of eight influenza seasons.

## Conclusions

This study showed that influenza vaccination in previous seasons may retain an important protective effect and is often a relevant effect modifier of the current-season IVE estimates. Moreover, most analyses that did not consider vaccination in previous seasons underestimated the EIV. Combinations of influenza vaccinations in the current and at least three previous seasons should be analysed to evaluate the existence of a modifying effect. When this is ruled out, a summarised analysis including vaccination status in three categories (current season, previous seasons only and unvaccinated) is a simple option to obtain adjusted estimates and to detect the effect of previous season vaccination. As most previous reports of IVE have not considered vaccination history, the benefit of influenza vaccination may be higher than reported in the literature.

## Supplementary Data

386000 Supplement
