# Supplementary material for: Simple models to include influenza vaccination history when evaluating the effect of influenza vaccination
Source: Euro Surveill. 2021 Aug 12;26(32):2001099. doi: 10.2807/1560-7917.ES.2021.26.32.2001099 (PMC8365179; doi:10.2807/1560-7917.ES.2021.26.32.2001099)
Supplement: Supplement [file 20-01099_CATALAN_Supplement.pdf]

"This supplementary material is hosted by Eurosurveillance as supporting information alongside the article [Management of influenza vaccination history in evaluating the effect of influenza vaccination], on behalf of the authors, who remain responsible for the accuracy and appropriateness of the content. The same standards for ethics, copyright, attributions and permissions as for the article apply. Supplements are not edited by Eurosurveillance and the journal is not responsible for the maintenance of any links or email addresses provided therein."

Supplementary Table S1. Percentage of virus (sub-)type by influenza season in Navarre, Spain, 2011/12 to 2018/19

Influenza season	A/H3N2	A/H1N1	B
2011/12	93.7	0.3	6.0
2012/13	6.0	25.4	68.6
2013/14	61.2	38.8	0.0
2014/15	50.9	1.9	47.2
2015/16	8.3	74.6	17.1
2016/17	98.6	0.0	1.4
2017/18	29.5	6.5	64.0
2018/19	47.0	52.5	0.6
Total	47.1	26.2	26.7

Supplementary Table S2. Sensitivity analysis of effect of current season influenza vaccination and of vaccination history including the diagnoses of influenza-like illness during the preceding season, Navarre, Spain, pooled analysis of the 2011/12 to 2018/19 (n=10,356)

Vaccination status and ILI diagnoses in the preceding season	No. of cases / controls	Vaccination effect, % (95% CI) [a]	p value
**Primary healthcare patients**			
**Vaccination status**			
Never vaccinated	2345/1135	0 (ref)	
1–2 prior and no current	103/79	37 (14 to 54)	0.004
3–5 prior and no current	26/31	63 (35 to 78)	<0.001
Current and no prior	53/59	59 (40 to 73)	<0.001
1–2 prior and current	67/58	46 (21 to 63)	0.001
3–5 prior and current	278/178	29 (8 to 45)	0.009
**ILI diagnosis in the preceding season**	112/107	49 (32 to 62)	<0.001
**Hospitalized patients**			
**Vaccination status**			
Never vaccinated	744/1094	0 (ref)	
1–2 prior and no current	134/257	25 (5 to 41)	0.017
3–5 prior and no current	148/308	39 (24 to 52)	<0.001
Current and no prior	49/122	39 (13 to 57)	0.007
1–2 prior and current	98/231	42 (24 to 56)	<0.001
3–5 prior and current	896/1863	39 (29 to 47)	<0.001
**ILI diagnosis in the preceding season**	23/60	24 (-22 to 55)	0.239
**Influenza A/H1N1**			
**Vaccination status**			
Never vaccinated	902/1507	0 (ref)	
1–2 prior and no current	72/228	24 (-5 to 45)	0.094
3–5 prior and no current	38/233	46 (20 to 64)	0.002
Current and no prior	32/127	45 (14 to 65)	0.009
1–2 prior and current	28/216	62 (41 to 76)	<0.001
3–5 prior and current	207/1509	53 (41 to 63)	<0.001
**ILI diagnosis in the preceding season**	35/113	55 (30 to 71)	<0.001
**Influenza A/H3N2**			
**Vaccination status**			
Never vaccinated	1280/2060	0 (ref)	
1–2 prior and no current	115/323	21 (-2 to 38)	0.071
3–5 prior and no current	87/333	39 (19 to 54)	0.001
Current and no prior	52/177	36 (8 to 55)	0.014
1–2 prior and current	90/277	27 (3 to 45)	0.027
3–5 prior and current	681/1987	22 (8 to 33)	0.003
**ILI diagnosis in the preceding season**	56/150	43 (19 to 60)	0.001
**Influenza B**			
**Vaccination status**			
Never vaccinated	869/1277	0 (ref)	
1–2 prior and no current	49/175	48 (25 to 64)	<0.001
3–5 prior and no current	46/164	38 (9 to 58)	0.015
Current and no prior	18/94	72 (52 to 84)	<0.001
1–2 prior and current	46/157	49 (25 to 65)	0.001
3–5 prior and current	273/978	49 (36 to 60)	<0.001
**ILI diagnosis in the preceding season**	43/97	32 (-3 to 55)	0.069

CI: confidence interval; ILI: influenza-like illness.
[a] Vaccination effect adjusted by age groups (9–44, 45–64, 65–84 and ≥85 years), major chronic conditions, healthcare setting (primary healthcare and hospital), and month-season of sample collection. Influenza A/H1N1 analysis includes 2012/13, 2013/14, 2015/16, 2017/18 and 2018/19 seasons. Influenza A/H3N2 analysis includes 2011/12, 2013/14, 2014/15, 2015/16, 2016/17, 2017/18 and 2018/19 seasons. Influenza B analysis includes 2011/12, 2012/13, 2014/15, 2015/16 and 2017/18 seasons.

Supplementary Table S3. Effect of current season influenza vaccination and of vaccination history by age group, Navarre, Spain, pooled analysis of the 2011/12 to 2018/19 (n=10,356)

	Aged 9–64 years			Aged ≥65 years		
	No. of cases / controls	Vaccination effect, % (95% CI) [a]	p value	No. of cases / controls	Vaccination effect, % (95% CI) [a]	p value
**Only current vaccination**						
Unvaccinated	2820/1998	0 (ref)		680/906	0 (ref)	
Vaccinated	306/475	42 (31 to 51)	<0.001	1135/2036	24 (13 to 33)	<0.001
**Summarized model with 1 prior season**						
Never vaccinated	2762/1900	0 (ref)		571/675	0 (ref)	
Prior and no current	58/98	35 (6 to 55)	0.020	109/231	40 (22 to 54)	<0.001
Current regardless prior	306/475	43 (32 to 52)	<0.001	1135/2036	32 (21 to 41)	<0.001
**Full model with 1 prior season**						
Never vaccinated	2762/1900	0 (ref)		571/675	0 (ref)	
Prior and no current	58/98	35 (6 to 55)	0.022	109/231	40 (22 to 54)	<0.001
Current and no prior	93/165	52 (37 to 64)	<0.001	135/234	29 (9 to 45)	0.008
Current and prior	213/310	37 (23 to 49)	<0.001	1000/1802	32 (22 to 41)	<0.001
**Summarized model with 3 prior seasons**						
Never vaccinated	2692/1798	0 (ref)		487/487	0 (ref)	
Any prior and no current	128/200	37 (19 to 51)	<0.001	193/353	34 (18 to 47)	<0.001
Current regardless prior	306/475	46 (34 to 53)	<0.001	1135/2036	34 (23 to 44)	<0.001
**Full model with 3 prior seasons**						
Never vaccinated	2692/1798	0 (ref)		487/553	0 (ref)	
1–2 prior and no current	112/163	34 (13 to 50) [b]	0.003	123/199	25 (1 to 42)	0.040
3 prior and no current	16/37	53 (11 to 75)	0.020	70/154	46 (26 to 61)	<0.001
Current and no prior	63/120	59 (42 to 70)	<0.001	55/95	36 (7 to 56)	0.019
1–2 prior and current	105/170	45 (27 to 58)	<0.001	247/461	36 (21 to 48)	<0.001
3 prior and current	138/185	34 (15 to 49) [b]	0.001	833/1480	34 (22 to 43)	<0.001
**Summarized model with 5 prior seasons**						
Never vaccinated	2647/1733	0 (ref)		442/496	0 (ref)	
Any prior and no current	173/265	39 (24 to 51)	<0.001	238/410	31 (14 to 45)	0.001
Current regardless prior	306/475	45 (35 to 54)	<0.001	1135/2036	35 (24 to 44)	<0.001
**Full model with 5 prior seasons**						
Never vaccinated	2647/1733	0 (ref)		442/496	0 (ref)	
1–2 prior and no current	130/184	36 (17 to 50)	0.001	107/152	19 (-8 to 40)	0.154
3–5 prior and no current	43/81	46 (19 to 64)	0.003	131/258	38 (20 to 52)	<0.001
Current and no prior	60/108	58 (40 to 70)	<0.001	42/73	35 (1 to 58)	0.045
1–2 prior and current	72/122	51 (33 to 65)	<0.001	93/167	36 (14 to 52)	0.003
3–5 prior and current	174/245	36 (20 to 49) [b]	<0.001	1000/1796	35 (23 to 44)	<0.001

CI: confidence interval.
[a] Vaccination effect adjusted by age groups (9–44, 45–64, 65–84 and ≥85 years), major chronic conditions, healthcare setting (primary healthcare and hospital), and month-season of sample collection.
[b] P-value<0.05 for comparison with the category of current season vaccination and no prior doses.

Supplementary Table S4. Effect of influenza vaccination in the current and prior seasons by (sub)type of influenza virus, Navarre, Spain, pooled analysis of the 2011/12 to 2018/19 (n=10,356)

	Influenza A/H1N1		Influenza A/H3N2		Influenza B	
	No. of cases / controls	Vaccination effect, % (95% CI) [a]	No. of cases / controls	Vaccination effect, % (95% CI) [a]	No. of cases / controls	Vaccination effect, % (95% CI)[a]
**Only-current-season model**						
Unvaccinated	1012/1968	0 (ref)	1482/2716	0 (ref)	964/1616	0 (ref)
Vaccinated	267/1852	47 (36 to 56)	823/2441	14 (2 to 25)	337/1229	44 (32 to 53)
**Summarized model with 1 prior season**						
Never vaccinated	971/1741	0 (ref)	1407/2391	0 (ref)	915/1447	0 (ref)
Prior and no current	41/227	40 (12 to 59)	75/325	43 (24 to 57)	49/169	29 (–2 to 51)
Current regardless prior	267/1852	51 (40 to 59)	823/2441	21 (9 to 32)	337/1229	47 (35 to 56)
**Full model with 1 prior season**						
Never vaccinated	971/1741	0 (ref)	1407/2391	0 (ref)	915/1447	0 (ref)
Prior and no current	41/227	41 (13 to 60)	75/325	43 (24 to 57)	49/169	29 (–3 to 51) [b]
Current and no prior	61/288	37 (13 to 55)	119/387	28 (8 to 43)	48/202	60 (42 to 72)
Current and prior	206/1564	54 (43 to 63)	704/2054	20 (7 to 31)	289/1027	43 (29 to 54)
**Summarized model with 3 prior seasons**						
Never vaccinated	921/1596	0 (ref)	1333/2177	0 (ref)	887/1342	0 (ref)
Any prior and no current	91/372	25 (1 to 44)	149/539	38 (23 to 51)	77/274	39 (17 to 55)
Current regardless prior	267/1852	50 (39 to 60)	823/2441	24 (13 to 35)	337/1229	50 (39 to 59)
**Full model with 3 prior seasons**						
Never vaccinated	921/1596	0 (ref)	1333/2177	0 (ref)	887/1342	0 (ref)
1-2 prior and no current	75/241	11 (–22 to 35)	106/350	33 (13 to 48)	52/178	35 (7 to 55) [b]
3 prior and no current	16/131	57 (24 to 76)	43/189	47 (24 to 64)	25/96	43 (6 to 65) [b]
Current and no prior	35/153	45 (16 to 64)	62/210	36 (11 to 54)	21/114	73 (55 to 84)
1-2 prior and current	64/470	55 (39 to67)	194/609	27 (10 to 40)	92/309	44 (25 to 58) [b]
3 prior and current	168/1229	51 (37 to 61)	567/1622	22 (8 to 33)	224/806	47 (33 to 58) [b]
**Summarized model with 5 prior seasons**						
Never vaccinated	902/1507	0 (ref)	1280/2060	0 (ref)	869/1277	0 (ref)
Any prior and no current	110/461	33 (13 to 49)	202/656	30 (15 to 43)	95/339	45 (27 to 58)
Current regardless prior	267/1852	53 (42 to 61)	823/2441	24 (11 to 34)	337/1229	53 (42 to 61)
**Full model with 5 prior seasons**						
Never vaccinated	902/1507	0 (ref)	1280/2060	0 (ref)	869/1277	0 (ref)
1-2 prior and no current	72/228	24 (–4 to 45)	115/323	21 (–1 to 39)	49/175	48 (25 to 64)
3-5 prior and no current	38/233	45 (19 to 63)	87/333	39 (19 to 54)	46/164	38 (9 to 58)
Current and no prior	32/127	45 (13 to 65)	52/177	36 (9 to 55)	18/94	72 (52 to 84)
1-2 prior and current	28/216	63 (42 to 76)	90/277	27 (4 to 45)	46/157	49 (26 to 65)
3-5 prior and current	207/1509	53 (40 to 62)	681/1987	22 (8 to 33)	273/978	49 (36 to 60) [b]

CI: confidence interval.

[a] Vaccination effect adjusted by age groups (9-44, 45-64, 65-84 and ≥85 years), major chronic conditions, healthcare setting (primary healthcare and hospital), and month-season of sample collection. Influenza A/H1N1 analysis includes 2012/13, 2013/14, 2015/16, 2017/18 and 2018/19 seasons. Influenza A/H3N2 analysis includes 2011/12, 2013/14, 2014/15, 2015/16, 2016/17, 2017/18 and 2018/19 seasons. Influenza B analysis includes 2011/12, 2012/13, 2014/15, 2015/16 and 2017/18 seasons.

[b] P-value <0.05 for comparison with the current season vaccination and no prior doses.

Supplementary Table S5. Effect of current season influenza vaccination and of vaccination history by age group in preventing primary healthcare consultations for laboratory-confirmed influenza, Navarre, Spain, pooled analysis of the 2011/12 to 2018/19 (n=4,412)

	Aged 9–64 years			Aged ≥65 years		
	No. of cases / controls	Vaccination effect, % (95% CI) [a]	p value	No. of cases / controls	Vaccination effect, % (95% CI) [a]	p value
**Only current vaccination**						
Unvaccinated	2330/1169	0 (ref)		144/76	0 (ref)	
Vaccinated	195/165	41 (26 to 53)	<0.001	230/130	18 (–22 to 45)	0.321
**Summarized model with 1 prior season**						
Never vaccinated	2297/1144	0 (ref)		136/66	0 (ref)	
Prior and no current	33/25	34 (–13 to 62)	0.130	8/10	58 (–18 to 85)	0.100
Current regardless prior	195/165	42 (26 to 54)	<0.001	230/130	25 (–13 to 51)	0.171
**Full model with 1 prior season**						
Never vaccinated	2297/1144	0 (ref)		136/66	0 (ref)	
Prior and no current	33/25	34 (–14 to 62)	0.136	8/10	58 (–18 to 85)	0.099
Current and no prior	62/70	57 (39 to 70)	<0.001	23/14	30 (–54 to 68)	0.376
Current and prior	133/95	30 (6 to 47)	0.018	180/116	25 (–16 to 51)	0.194
**Summarized model with 3 prior seasons**						
Never vaccinated	2263/1107	0 (ref)		122/61	0 (ref)	
Any prior and no current	67/62	50 (28 to 65)	<0.001	22/15	21 (–74 to 64)	0.557
Current regardless prior	195/165	43 (28 to 55)	<0.001	203/130	22 (–20 to 50)	0.257
**Full model with 3 prior seasons**						
Never vaccinated	2263/1107	0 (ref)		122/61	0 (ref)	
1–2 prior and no current	61/52	46 (20 to 63) [b]	0.002	18/9	–5 (–170 to 56)	0.863
3 prior and no current	6/10	71 (16 to 90)	0.023	4/6	66 (–36 to 92)	0.127
Current and no prior	44/56	67 (45 to 76)	<0.001	14/9	33 (–73 to 74)	0.406
1–2 prior and current	70/55	37 (7 to 57) [b]	0.019	38/27	33 (–28 to 65)	0.221
3 prior and current	81/54	28 (–5 to 50) [b]	0.086	151/94	17 (–32 to 48)	0.426
**Summarized model with 5 prior seasons**						
Never vaccinated	2231/1081	0 (ref)		114/54	0 (ref)	
Any prior and no current	99/88	47 (28 to 61)	<0.001	30/22	39 (–22 to 69)	0.165
Current regardless prior	195/165	44 (29 to 55)	<0.001	203/130	29 (–12 to 55)	0.140
**Full model with 5 prior seasons**						
Never vaccinated	2231/1081	0 (ref)		114/54	0 (ref)	
1–2 prior and no current	80/66	41 (17 to 59)	0.002	23/13	12 (–75 to 65)	0.552
3–5 prior and no current	19/22	63 (29 to 80)	0.003	7/9	63 (–13 to 88)	0.080
Current and no prior	43/51	61 (40 to 74)	<0.001	10/8	53 (–35 to 84)	0.160
1–2 prior and current	52/44	44 (15 to 64)	0.007	15/14	52 (–13 to 80)	0.093
3–5 prior and current	100/70	30 (3 to 50) [b]	0.003	178/108	23 (–22 to 52)	0.267

CI: confidence interval.
[a] Vaccination effect adjusted by age groups (9–44, 45–64, 65–84 and ≥85 years), major chronic conditions, and month-season of sample collection.
[b] P-value<0.05 for comparison with the category of current season vaccination and no prior doses.

Supplementary Table S6. Effect of current season influenza vaccination and of vaccination history by age group in preventing hospitalization with laboratory-confirmed influenza, Navarre, Spain, pooled analysis of the 2011/12 to 2018/19 (n=5,944)

	Aged 9–64 years			Aged ≥65 years		
	No. of cases / controls	Vaccination effect, % (95% CI) [a]	p value	No. of cases / controls	Vaccination effect, % (95% CI) [a]	p value
**Only current vaccination**						
Unvaccinated	490/829	0 (ref)		536/830	0 (ref)	
Vaccinated	111/310	44 (26 to 57)	<0.001	932/1906	24 (13 to 34)	<0.001
**Summarized model with 1 prior season**						
Never vaccinated	465/756	0 (ref)		435/609	0 (ref)	
Prior and no current	25/75	34 (–9 to 60)	0.105	101/221	39 (20 to 54)	<0.001
Current regardless prior	111/310	46 (29 to 58)	<0.001	932/1906	33 (22 to 42)	<0.001
**Full model with 1 prior season**						
Never vaccinated	465/756	0 (ref)		435/609	0 (ref)	
Prior and no current	25/73	34 (–9 to 60)	0.105	101/221	39 (20 to 54)	<0.001
Current and no prior	31/95	46 (16 to 66)	0.007	112/220	29 (7 to 46)	0.012
Current and prior	80/215	45 (26 to 60)	<0.001	820/1686	33 (22 to 43)	<0.001
**Summarized model with 3 prior seasons**						
Never vaccinated	429/691	0 (ref)		365/492	0 (ref)	
Any prior and no current	61/138	25 (–7 to 48)	0.108	171/338	36 (18 to 49)	<0.001
Current regardless prior	111/310	47 (30 to 60)	<0.001	932/1906	36 (24 to 45)	<0.001
**Full model with 3 prior seasons**						
Never vaccinated	429/691	0 (ref)		365/492	0 (ref)	
1–2 prior and no current	51/111	22 (–15 to 47)	0.212	105/190	27 (2 to 45)	0.033
3 prior and no current	10/27	40 (–32 to 83)	0.205	66/148	46 (25 to 61)	<0.001
Current and no prior	19/64	50 (12 to 71)	0.017	41/86	36 (4 to 58)	0.030
1–2 prior and current	35/115	53 (28 to 69)	0.001	209/434	37 (21 to 49)	<0.001
3 prior and current	57/131	40 (14 to 59)	0.005	682/1386	35 (23 to 46)	<0.001
**Summarized model with 5 prior seasons**						
Never vaccinated	416/652	0 (ref)		328/442	0 (ref)	
Any prior and no current	74/177	32 (5 to 51)	0.021	208/388	30 (12 to 45)	0.002
Current regardless prior	111/310	48 (32 to 61)	<0.001	932/1906	35 (23 to 46)	<0.001
**Full model with 5 prior seasons**						
Never vaccinated	416/652	0 (ref)		328/442	0 (ref)	
1–2 prior and no current	50/118	31 (–1 to 53)	0.057	84/139	17 (–15 to 39)	0.259
3–5 prior and no current	24/59	33 (–13 to 61)	0.132	124/249	38 (18 to 52)	0.001
Current and no prior	17/57	52 (13 to 73)	0.016	32/65	31 (–10 to 56)	0.117
1–2 prior and current	20/78	60 (31 to 77)	0.001	78/153	33 (9 to 51)	0.012
3–5 prior and current	74/175	43 (20 to 59)	0.001	822/1688	36 (24 to 46)	<0.001

CI: confidence interval.
[a] Vaccination effect adjusted by age groups (9–44, 45–64, 65–84 and ≥85 years), major chronic conditions, and month-season of sample collection.

Supplementary Table S7. Effect of influenza vaccination in the current and prior seasons in preventing primary healthcare consultations for laboratory-confirmed influenza by (sub)type of influenza virus, Navarre, Spain, pooled analysis of the 2011/12 to 2018/19 (n=4,412)

	Influenza A/H1N1		Influenza A/H3N2		Influenza B	
	No. of cases / controls	Vaccination effect, % (95% CI) [a]	No. of cases / controls	Vaccination effect, % (95% CI) [a]	No. of cases / controls	Vaccination effect, % (95% CI)[a]
**Only-current-season model**						
Unvaccinated	677/751	0 (ref)	1067/1106	0 (ref)	701/805	0 (ref)
Vaccinated	78/197	39 (12 to 58)	221/274	23 (2 to 40)	101/171	45 (24 to 61)
**Summarized model with 1 prior season**						
Never vaccinated	663/733	0 (ref)	1049/1071	0 (ref)	692/785	0 (ref)
Prior and no current	14/18	11 (–91 to 59)	18/35	51 (8 to 74)	9/20	41 (–41 to 76)
Current regardless prior	78/197	39 (12 to 58)	221/274	26 (5 to 42)	101/171	47 (25 to 62)
**Full model with 1 prior season**						
Never vaccinated	633/733	0 (ref)	1049/1071	0 (ref)	692/785	0 (ref)
Prior and no current	14/18	12 (–89 to 59)	18/35	50 (6 to 73)	9/20	41 (–43 to 75)
Current and no prior	27/56	27 (–28 to 58)	37/81	53 (26 to 69)	21/53	65 (37 to 80)
Current and prior	51/141	45 (15 to 65)	184/193	12 (–17 to 34)[b]	80/118	37 (7 to 57)
**Summarized model with 3 prior seasons**						
Never vaccinated	646/708	0 (ref)	1206/1034	0 (ref)	684/763	0 (ref)
Any prior and no current	31/43	25 (–26 to 55)	41/72	51 (25 to 68)	17/42	49 (4 to 73)
Current regardless prior	78/197	40 (14 to 59)	221/274	29 (8 to 45)	101/171	48 (37 to 63)
**Full model with 3 prior seasons**						
Never vaccinated	646/708	0 (ref)	1026/1034	0 (ref)	684/763	0 (ref)
1-2 prior and no current	29/36	19 (–40 to 53)	36/56	43 (10 to 64)	14/30	46 (–9 to 73)
3 prior and no current	2/7	65 (–91 to 94)	5/16	73 (18 to 91)	3/12	61 (–70 to 91)
Current and no prior	19/40	41 (–12 to 69)	25/62	60 (32 to 76)	14/41	70 (41 to 85)
1-2 prior and current	23/61	31 (–24 to 61)	51/76	38 (7 to 58)	34/43	29 (–20 to 58) [b]
3 prior and current	36/96	47 (10 to 68)	145/136	3 (–34 to 30) [b]	53/87	46 (14 to 65)
**Summarized model with 5 prior seasons**						
Never vaccinated	638/688	0 (ref)	1002/1004	0 (ref)	676/738	0 (ref)
Any prior and no current	39/63	30 (–11 to 56)	65/102	42 (17 to 59)	25/67	60 (34 to 76)
Current regardless prior	78/197	41 (15 to 59)	221/274	29 (9 to 45)	101/171	50 (30 to 65)
**Full model with 5 prior seasons**						
Never vaccinated	638/688	0 (ref)	1002/1004	0 (ref)	676/738	0 (ref)
1-2 prior and no current	32/50	27 (–21 to 56)	53/72	21 (–1 to 39)	18/47	63 (34 to 79)
3-5 prior and no current	7/13	42 (–59 to 79)	12/30	39 (19 to 54)	7/20	49 (–36 to 81)
Current and no prior	18/38	40 (–16 to 69)	23/56	36 (9 to 55)	12/38	72 (52 to 86)
1-2 prior and current	14/39	38 (–27 to 69)	33/52	27 (4 to 45)	20/31	46 (–1 to 72)
3-5 prior and current	46/120	43 (8 to 65)	165/166	22 (8 to 33) [b]	69/102	41 (10 to 61)

CI: confidence interval.

[a] Vaccination effect adjusted by age groups (9-44, 45-64, 65-84 and ≥85 years), major chronic conditions, and month-season of sample collection. Influenza A/H1N1 analysis includes 2012/13, 2013/14, 2015/16, 2017/18 and 2018/19 seasons. Influenza A/H3N2 analysis includes 2011/12, 2013/14, 2014/15, 2015/16, 2016/17, 2017/18 and 2018/19 seasons. Influenza B analysis includes 2011/12, 2012/13, 2014/15, 2015/16 and 2017/18 seasons.

[b] P-value <0.05 for comparison with the current season vaccination and no prior doses.

Supplementary Table S8. Effect of influenza vaccination in the current and prior seasons in preventing hospitalization with laboratory-confirmed influenza by (sub)type of influenza virus, Navarre, Spain, pooled analysis of the 2011/12 to 2018/19 (n=5,944)

	Influenza A/H1N1		Influenza A/H3N2		Influenza B	
	No. of cases / controls	Vaccination effect, % (95% CI) [a]	No. of cases / controls	Vaccination effect, % (95% CI) [a]	No. of cases / controls	Vaccination effect, % (95% CI)[a]
**Only-current-season model**						
Unvaccinated	335/1217	0 (ref)	415/1610	0 (ref)	263/811	0 (ref)
Vaccinated	189/1655	49 (36 to 59)	602/2167	9 (–7 to 23)	236/1058	43 (39 to 55)
**Summarized model with 1 prior season**						
Never vaccinated	308/1008	0 (ref)	358/1320	0 (ref)	223/662	0 (ref)
Prior and no current	27/209	47 (16 to 66)	57/290	41 (18 to 57)	40/149	27 (–11 to 52)
Current regardless prior	189/1655	54 (42 to 64)	602/2167	19 (3 to 31)	236/1058	47 (32 to 59)
**Full model with 1 prior season**						
Never vaccinated	308/1008	0 (ref)	358/1320	0 (ref)	223/662	0 (ref)
Prior and no current	27/209	47 (16 to 67)	57/290	41 (18 to 57) [b]	40/149	27 (–11 to 52)
Current and no prior	34/232	41 (10 to 61)	82/306	12 (–18 to 37)	27/149	57 (32 to 73)
Current and prior	155/1423	57 (44 to 67)	520/1861	20 (4 to 33)	209/909	45 (29 to 58)
**Summarized model with 3 prior seasons**						
Never vaccinated	275/888	0 (ref)	307/1143	0 (ref)	203/579	0 (ref)
Any prior and no current	60/329	26 (–4 to 48)	108/467	34 (13 to 49)	60/232	37 (9 to 56)
Current regardless prior	189/1655	53 (40 to 64) [b]	602/2167	21 (5 to 34)	236/1058	52 (37 to 63)
**Full model with 3 prior seasons**						
Never vaccinated	275/888	0 (ref)	307/1143	0 (ref)	203/579	0 (ref)
1-2 prior and no current	46/205	8 (–37 to 38)	70/294	28 (2 to 47)	38/148	32 (–4 to 56) [b]
3 prior and no current	14/124	56 (19 to 76)	38/173	43 (15 to 62)	22/84	44 (3 to 67)
Current and no prior	16/113	47 (6 to 71)	37/148	9 (–37 to 39)	7/73	78 (49 to 90)
1-2 prior and current	41/409	60 (41 to 73)	143/533	22 (0 to 39)	58/266	50 (28 to 65)
3 prior and current	132/1133	52 (37 to 64)	422/1486	23 (6 to 37)	171/719	49 (33 to 62) [b]
**Summarized model with 5 prior seasons**						
Never vaccinated	264/819	0 (ref)	278/1056	0 (ref)	193/539	0 (ref)
Any prior and no current	71/398	36 (12 to 54)	137/554	24 (2 to 41)	70/272	37 (11 to 56)
Current regardless prior	189/1655	56 (44 to 66) [b]	602/2167	19 (2 to 33)	236/1058	53 (38 to 64)
**Full model with 5 prior seasons**						
Never vaccinated	264/819	0 (ref)	278/1056	0 (ref)	193/539	0 (ref)
1-2 prior and no current	40/178	24 (–16 to 49)	62/251	14 (–19 to 38)	31/128	36 (–1 to 59)
3-5 prior and no current	31/220	48 (18 to 66)	75/303	31 (6 to 50)	39/144	38 (4 to 60)
Current and no prior	14/89	47 (2 to 72)	29/121	11 (–40 to 43)	6/56	74 (20 to 70)
1-2 prior and current	14/177	70 (45 to 83)	57/225	14 (–21 to 49)	26/126	51 (20 to 70)
3-5 prior and current	161/1389	56 (42 to 66)	516/1821	21 (3 to 35)	204/876	52 (36 to 63)

CI: confidence interval.
[a] Vaccination effect adjusted by age groups (9-44, 45-64, 65-84 and ≥85 years), major chronic conditions, and month-season of sample collection. Influenza A/H1N1 analysis includes 2012/13, 2013/14, 2015/16, 2017/18 and 2018/19 seasons. Influenza A/H3N2 analysis includes 2011/12, 2013/14, 2014/15, 2015/16, 2016/17, 2017/18 and 2018/19 seasons. Influenza B analysis includes 2011/12, 2012/13, 2014/15, 2015/16 and 2017/18 seasons.
[b] P-value <0.05 for comparison with the current season vaccination and no prior doses.

Supplementary Table S9a. Effect of current season influenza vaccination and of vaccination history by influenza season, Navarre, Spain

	2011/12 season			2012/13 season		
	No. of cases / controls	Vaccination effect, % (95% CI) [a]	p value	No. of cases / controls	Vaccination effect, % (95% CI) [a]	p value
**Only-current-season model**						
Unvaccinated	302/188	0 (ref)		293/188	0 (ref)	
Vaccinated	49/47	20 (-47 to 57)	0.467	22/70	69 (41 to 83)	<0.001
**Summarized model with 1 prior season**						
Never vaccinated	299/177	0 (ref)		284/184	0 (ref)	
Prior and no current	3/11	81 (20 to 95)	0.023	9/4	-327 (-1712 to -1)	0.049
Current regardless prior	49/47	29 (-32 to 62)	0.280	22/70	66 (36 to 82)	0.001
**Full model with 1 prior season**						
Never vaccinated	299/177	0 (ref)		284/184	0 (ref)	
Prior and no current	3/11	81 (20 to 95)	0.023	9/4	-326 (-1712 to 0)	0.049
Current and no prior	6/7	34 (-165 to 83)	0.562	4/12	64 (-21 to 89)	0.097
Current and prior	43/40	28 (-37 to 62)	0.317	18/58	67 (32 to 84)	0.003
**Summarized model with 3 prior seasons**						
Never vaccinated	293/167	0 (ref)		279/174	0 (ref)	
Any prior and no current	9/21	66 (15 to 86)	0.020	14/14	2 (-135 to 59)	0.965
Current regardless prior	49/47	33 (-26 to 64)	0.218	22/70	69 (41 to 84)	<0.001
**Full model with 3 prior seasons**						
Never vaccinated	293/167	0 (ref)		279/174	0 (ref)	
1–2 prior and no current	7/15	61 (-11 to 86)	0.078	10/12	29 (-85 to 73)	0.482
3 prior and no current	2/6	77 (-32 to 96)	0.099	4/2	-307 (-2867 to 44)	0.166
Current and no prior	3/4	18 (-511 to 89)	0.847	1/5	86 (-26 to 98)	0.079
1–2 prior and current	13/10	-3 (-204 to 65)	0.959	7/22	65 (8 to 87)	0.034
3 prior and current	33/33	43 (-16 to 72)	0.118	14/43	66 (24 to 85)	0.009
**Summarized model with 5 prior seasons**						
Never vaccinated	289/164	0 (ref)		276/169	0 (ref)	
Any prior and no current	13/24	60 (12 to 82)	0.023	17/19	22 (-68 to 64)	0.525
Current regardless prior	49/47	33 (-25 to 64)	0.208	22/70	70 (43 to 84)	<0.001
**Full model with 5 prior seasons**						
Never vaccinated	289/164	0 (ref)		276/169	0 (ref)	
1–2 prior and no current	9/14	44 (-50 to 79)	0.250	10/13	44 (-39 to 77)	0.213
3–5 prior and no current	4/10	78 (20 to 94)	0.022	7/6	-63 (-500 to 56)	0.463
Current and no prior	3/4	20 (-500 to 89)	0.831	1/4	84 (-53 to 98)	0.112
1–2 prior and current	4/6	48 (-140 to 89)	0.404	4/12	70 (-1 to 91)	0.051
3–5 prior and current	42/37	32 (-35 to 65)	0.272	17/54	67 (30 to 84)	0.004

CI: confidence interval.
[a] Vaccination effect adjusted by age groups (9–44, 45–64, 65–84 and ≥85 years), major chronic conditions, healthcare setting (primary healthcare and hospital), and month of sample collection.

Supplementary Table S9b. Effect of current season influenza vaccination and of vaccination history by influenza season, Navarre, Spain

	2013/14 season			2014/15 season		
	No. of cases / controls	Vaccination effect, % (95% CI) [a]	p value	No. of cases / controls	Vaccination effect, % (95% CI) [a]	p value
**Only-current-season model**						
Unvaccinated	385/287	0 (ref)		423/307	0 (ref)	
Vaccinated	142/189	28 (0 to 49)	0.049	150/175	15 (-19 to 39)	0.350
**Summarized model with 1 prior season**						
Never vaccinated	362/265	0 (ref)		408/274	0 (ref)	
Prior and no current	23/22	11 (-74 to 54)	0.733	15/33	54 (9 to 77)	0.026
Current regardless prior	142/189	30 (0 to 50)	0.047	150/175	24 (-8 to 47)	0.121
**Full model with 1 prior season**						
Never vaccinated	362/265	0 (ref)		408/274	0 (ref)	
Prior and no current	23/22	11 (-73 to 55)	0.724	15/33	54 (9 to 77)	0.025
Current and no prior	23/32	21 (-46 to 57)	0.453	21/20	9 (-80 to 54)	0.781
Current and prior	119/157	32 (1 to 53)	0.043	129/155	27 (-6 to 50)	0.096
**Summarized model with 3 prior seasons**						
Never vaccinated	338/245	0 (ref)		397/257	0 (ref)	
Any prior and no current	47/42	2 (-64 to 41)	0.941	26/50	55 (21 to 74)	0.005
Current regardless prior	142/189	29 (-3 to 51)	0.067	150/175	29 (-2 to 50)	0.062
**Full model with 3 prior seasons**						
Never vaccinated	338/245	0 (ref)		397/257	0 (ref)	
1–2 prior and no current	36/31	-23 (-204 to 50)	0.654	21/32	45 (-1 to 71)	0.055
3 prior and no current	11/11	-26 (-249 to 55)	0.661	5/18	73 (23 to 91)	0.015
Current and no prior	10/12	-9 (-280 to 69)	0.893	5/13	73 (21 to 91)	0.018
1–2 prior and current	38/58	27 (-96 to 73)	0.532	53/38	-11 (-84 to 33) [b]	0.682
3 prior and current	94/119	6 (-140 to 63)	0.896	92/124	38 (6 to 59)	0.024
**Summarized model with 5 prior seasons**						
Never vaccinated	327/232	0 (ref)		383/238	0 (ref)	
Any prior and no current	58/55	6 (-52 to 42)	0.808	40/69	55 (28 to 72)	0.001
Current regardless prior	142/189	30 (-2 to 52)	0.063	150/175	34 (4 to 54)	0.028
**Full model with 5 prior seasons**						
Never vaccinated	327/232	0 (ref)		383/238	0 (ref)	
1–2 prior and no current	35/25	-19 (-120 to 35)	0.576	24/36	53 (17 to 74)	0.010
3–5 prior and no current	23/30	29 (-36 to 63)	0.297	16/33	59 (18 to 80)	0.012
Current and no prior	9/10	18 (-116 to 69)	0.688	5/9	61 (-25 to 88)	0.113
1–2 prior and current	18/20	34 (-36 to 68)	0.265	29/19	-14 (-111 to 40)	0.688
3–5 prior and current	115/159	32 (-3 to 55)	0.068	116/147	41 (11 to 61)	0.011

CI: confidence interval.
[a] Vaccination effect adjusted by age groups (9–44, 45–64, 65–84 and ≥85 years), major chronic conditions, healthcare setting (primary healthcare and hospital), and month of sample collection.
[b] P value < 0.05 for comparison with the category of current season vaccination and no prior doses.

Supplementary Table S9c. Effect of current season influenza vaccination and of vaccination history by influenza season, Navarre, Spain

	2015/16 season			2016/17 season		
	No. of cases / controls	Vaccination effect, % (95% CI) [a]	p value	No. of cases / controls	Vaccination effect, % (95% CI) [a]	p value
**Only-current-season model**						
Unvaccinated	582/398	0 (ref)		388/441	0 (ref)	
Vaccinated	152/305	42 (22 to 56)	<0.001	274/437	16 (-8 to 35)	0.170
**Summarized model with 1 prior season**						
Never vaccinated	555/364	0 (ref)		363/383	0 (ref)	
Prior and no current	27/34	14 (-52 to 51)	0.605	25/58	49 (13 to 70)	0.013
Current regardless prior	152/305	43 (23 to 58)	<0.001	274/437	25 (2 to 42)	0.034
**Full model with 1 prior season**						
Never vaccinated	555/364	0 (ref)		363/383	0 (ref)	
Prior and no current	27/34	12 (-57 to 50)	0.669	25/58	48 (12 to 69)	0.016
Current and no prior	26/53	55 (25 to 73)	0.003	42/84	41 (9 to 62)	0.016
Current and prior	126/252	38 (14 to 56)	0.005	232/353	20 (-7 to 39)	0.129
**Summarized model with 3 prior seasons**						
Never vaccinated	538/344	0 (ref)		340/331	0 (ref)	
Any prior and no current	44/54	18 (-31 to 49)	0.410	48/110	52 (28 to 68)	<0.001
Current regardless prior	152/305	44 (24 to 59)	<0.001	274/437	32 (11 to 49)	0.006
**Full model with 3 prior seasons**						
Never vaccinated	538/344	0 (ref)		340/331	0 (ref)	
1–2 prior and no current	34/33	-4 (-80 to 40)	0.880	35/74	46 (14 to 66)	0.010
3 prior and no current	10/21	46 (-23 to 76)	0.142	13/36	62 (25 to 81)	0.006
Current and no prior	15/35	65 (32 to 82)	0.002	24/45	50 (13 to 71)	0.014
1–2 prior and current	31/78	53 (24 to 71)	0.002	58/113	37 (6 to 58)	0.024
3 prior and current	106/192	32 (2 to 53)	0.036	192/279	26 (-1 to 45)	0.060
**Summarized model with 5 prior seasons**						
Never vaccinated	524/325	0 (ref)		331/320	0 (ref)	
Any prior and no current	58/73	22 (-19 to 49)	0.247	57/121	48 (23 to 65)	0.001
Current regardless prior	152/305	46 (26 to 60)	<0.001	274/437	32 (10 to 49)	0.007
**Full model with 5 prior seasons**						
Never vaccinated	524/325	0 (ref)		331/320	0 (ref)	
1–2 prior and no current	42/39	-2 (-68 to 38)	0.934	33/58	35 (-6 to 60)	0.087
3–5 prior and no current	16/34	45 (-7 to 72)	0.079	24/63	58 (28 to 76)	0.002
Current and no prior	13/32	69 (37 to 84)	0.001	21/41	50 (10 to 72)	0.022
1–2 prior and current	13/35	61 (22 to 80)	0.008	24/48	47 (9 to 70)	0.023
3–5 prior and current	126/238	36 (9 to 55)	0.014	229/348	26 (0 to 46)	0.050

CI: confidence interval.
[a] Vaccination effect adjusted by age groups (9–44, 45–64, 65–84 and ≥85 years), major chronic conditions, healthcare setting (primary healthcare and hospital), and month of sample collection.

Supplementary Table S9d. Effect of current season influenza vaccination and of vaccination history by influenza season, Navarre, Spain

	2017/18 season			2018/19 season		
	No. of cases / controls	Vaccination effect, % (95% CI) [a]	p value	No. of cases / controls	Vaccination effect, % (95% CI) [a]	p value
**Only-current-season model**						
Unvaccinated	647/535	0 (ref)		480/560	0 (ref)	
Vaccinated	397/632	38 (23 to 49)	<0.001	255/656	29 (10 to 44)	0.005
**Summarized model with 1 prior season**						
Never vaccinated	605/448	0 (ref)		457/480	0 (ref)	
Prior and no current	42/87	47 (20 to 65)	0.002	23/80	51 (18 to 71)	0.007
Current regardless prior	397/632	44 (31 to 55)	<0.001	255/656	36 (18 to 51)	<0.001
**Full model with 1 prior season**						
Never vaccinated	605/448	0 (ref)		457/480	0 (ref)	
Prior and no current	42/87	47 (20 to 65)	0.003	23/80	51 (18 to 71)	0.007
Current and no prior	67/110	50 (29 to 65)	<0.001	39/81	31 (-8 to 55)	0.104
Current and prior	330/522	43 (28 to 55)	<0.001	216/575	38 (19 to 52)	<0.001
**Summarized model with 3 prior seasons**						
Never vaccinated	568/400	0 (ref)		426/433	0 (ref)	
Any prior and no current	79/135	37 (12 to 55)	0.007	54/127	36 (6 to 57)	0.022
Current regardless prior	397/632	46 (32 to 57)	<0.001	255/656	37 (18 to 51)	<0.001
**Full model with 3 prior seasons**						
Never vaccinated	568/400	0 (ref)		426/433	0 (ref)	
1–2 prior and no current	53/86	30 (-5 to 53)	0.082	39/79	31 (-8 to 56)	0.101
3 prior and no current	26/49	49 (14 to 70)	0.011	15/48	44 (-5 to 70)	0.071
Current and no prior	37/57	52 (24 to 70)	0.002	23/44	33 (-18 to 62)	0.164
1–2 prior and current	101/161	44 (23 to 59)	<0.001	51/151	48 (23 to 65)	0.001
3 prior and current	259/414	45 (29 to 58)	<0.001	181/463	33 (10 to 50)	0.007
**Summarized model with 5 prior seasons**						
Never vaccinated	550/381	0 (ref)		409/400	0 (ref)	
Any prior and no current	97/154	36 (12 to 53)	0.006	71/160	33 (5 to 53)	0.023
Current regardless prior	397/632	46 (32 to 57)	<0.001	255/656	38 (19 to 52)	<0.001
**Full model with 5 prior seasons**						
Never vaccinated	550/381	0 (ref)		409/400	0 (ref)	
1–2 prior and no current	50/73	30 (-6 to 53)	0.090	34/78	41 (6 to 63)	0.025
3–5 prior and no current	47/81	40 (9 to 61)	0.015	37/82	24 (-20 to 52)	0.236
Current and no prior	30/45	53 (22 to 72)	0.004	20/36	31 (-28 to 63)	0.238
1–2 prior and current	48/85	51 (27 to 68)	0.001	25/64	47 (10 to 69)	0.019
3–5 prior and current	319/502	44 (28 to 57)	<0.001	210/556	36 (15 to 52)	0.002

CI: confidence interval.
[a] Vaccination effect adjusted by age groups (9–44, 45–64, 65–84 and ≥85 years), major chronic conditions, healthcare setting (primary healthcare and hospital), and month of sample collection.

Supplementary Table S10. Comparison of the effect estimates of influenza vaccination from different models with the estimate that compares people vaccinated in the current season with those unvaccinated in the current and five prior seasons, by influenza season, Navarre, Spain

Season and model	Vaccination effect, % (95% CI) [a]	Absolute difference of vaccination effect, %
**2011/12**		
Summarized model with 5 prior seasons	33 (-25 to 64)	Reference
Summarized model with 3 prior seasons	33 (-26 to 64)	0
Summarized model with 1 prior season	29 (-32 to 62)	-4
Only-current-season model	20 (-47 to 57)	-13 [b,c]
**2012/13**		
Summarized model with 5 prior seasons	70 (43 to 84)	Reference
Summarized model with 3 prior seasons	69 (41 to 84)	-1
Summarized model with 1 prior season	66 (36 to 82)	-4
Only-current-season model	69 (41 to 83)	-1
**2013/14**		
Summarized model with 5 prior seasons	30 (-2 to 52)	Reference
Summarized model with 3 prior seasons	29 (-3 to 51)	-1
Summarized model with 1 prior season	30 (0 to 50)	0
Only-current-season model	28 (0 to 49)	-2
**2014/15**		
Summarized model with 5 prior seasons	34 (4 to 54)	Reference
Summarized model with 3 prior seasons	29 (-2 to 50)	-5
Summarized model with 1 prior season	24 (-8 to 47)	-10 [b]
Only-current-season model	15 (-19 to 39)	-19 [b,c]
**2015/16**		
Summarized model with 5 prior seasons	46 (26 to 60)	Reference
Summarized model with 3 prior seasons	44 (24 to 59)	-2
Summarized model with 1 prior season	43 (23 to 58)	-3
Only-current-season model	42 (22 to 56)	-4
**2016/17**		
Summarized model with 5 prior seasons	32 (10 to 49)	Reference
Summarized model with 3 prior seasons	32 (11 to 49)	0
Summarized model with 1 prior season	25 (2 to 42)	-7 [b]
Only-current-season model	16 (-8 to 35)	-16 [b,c]
**2017/18**		
Summarized model with 5 prior seasons	46 (32 to 57)	Reference
Summarized model with 3 prior seasons	46 (32 to 57)	0
Summarized model with 1 prior season	44 (31 to 55)	-2
Only-current-season model	38 (23 to 49)	-8 [b]
**2018/19**		
Summarized model with 5 prior seasons	38 (19 to 52)	Reference
Summarized model with 3 prior seasons	37 (18 to 51)	-1
Summarized model with 1 prior season	36 (18 to 51)	-2
Only-current-season model	29 (10 to 44)	-9 [b]

CI, confidence interval.

[a] Vaccination effect adjusted by age groups (9–44, 45–64, 65–84 and ≥85 years), major chronic conditions, healthcare setting (primary healthcare and hospital), and month-season of sample collection.

[b] Absolute difference of vaccine effectiveness of ±5% or over was considered as a bias.

[c] Absolute difference of vaccine effect of ±10% or over was considered as a relevant bias.

Interaction assessment

As a complementary way of modifying effect evaluation interaction terms between current-season vaccination and prior doses received (0, 1–2 or 3–5 doses) were tested. Current season vaccination maintained a preventive effect as compared to unvaccinated individuals in the current and five prior seasons; however a negative interaction between influenza vaccination in the current and in prior seasons was observed in the overall analysis and in specific analyses of primary healthcare patients, hospitalized patients, patients younger than 65 years old, and against influenza A/H3N2 and influenza B.

Supplementary Table S11. Effect of current season influenza vaccination and of previous doses and the interaction terms between both, Navarre, Spain, pooled analysis of the 2011/12 to 2018/19 (n=10,356)

	Adjusted odds ratio (95% CI) [a]	Interaction odds ratio (95% CI) [a]	Interaction p value	Adjusted odds ratio (95% CI) [a]	Interaction odds ratio (95% CI) [a]	Interaction p value
	**All patients**			**Primary healthcare patients**		
**Current vaccination**	0.50 (0.38-0.65)			0.41 (0.28-0.61)		
**Number of prior doses**						
0 doses	1			1		
1–2 doses	0.70 (0.58–0.84)	1.59 (1.09–2.32)	0.016	0.63 (0.46–0.86)	2.11 (1.11–3.78)	0.022
3–5 doses	0.57 (0.47–0.71)	2.17 (1.54–3.04)	<0.001	0.37 (0.21–0.64)	4.77 (2.38–9.56)	<0.001
	**Hospital patients**			**Aged 9–64 years**		
**Current vaccination**	0.60 (0.42–0.86)			0.42 (0.30–0.60)		
**Number of prior doses**						
0 doses	1			1		
1–2 doses	0.75 (0.59–0.95)	1.29 (0.79–2.09)	0.309	0.64 (0.50–0.83)	1.79 (1.06–3.04)	0.031
3–5 doses	0.61 (0.48–0.77)	1.67 (1.10–2.53)	0.017	0.54 (0.36–0.81)	2.80 (1.58–4.95)	<0.001
	**Aged ≥65 years**			**Influenza A/H1N1**		
**Current vaccination**	0.65 (0.42–0.99)			0.55 (0.35–0.87)		
**Number of prior doses**						
0 doses	1			1		
1–2 doses	0.81 (0.60–1.08)	1.22 (0.70–2.15)	0.482	0.76 (0.55–1.04)	0.89 (0.45–1.77)	0.749
3–5 doses	0.62 (0.48–0.80)	1.63 (1.01–2.65)	0.047	0.55 (0.37–0.81)	1.57 (0.86–2.86)	0.143
	**Influenza A/H3N2**			**Influenza B**		
**Current vaccination**	0.64 (0.45–0.91)			0.28 (0.16–0.48)		
**Number of prior doses**						
0 doses	1			1		
1–2 doses	0.79 (0.61–1.01)	1.45 (0.88–2.38)	0.150	0.52 (0.36–0.75)	3.53 (1.68–7.41)	0.001
3–5 doses	0.61 (0.46–0.81)	2.30 (1.30–3.15)	0.006	0.62 (0.42–0.91)	2.99 (1.52–5.85)	<0.001

CI: confidence interval.
[a] Odds ratio adjusted by age groups (9–44, 45–64, 65–84 and ≥85 years), major chronic conditions, healthcare setting (primary healthcare and hospital), and month-season of sample collection. Influenza A/H1N1 analysis includes 2012/13, 2013/14, 2015/16, 2017/18 and 2018/19 seasons. Influenza A/H3N2 analysis includes 2011/12, 2013/14, 2014/15, 2015/16, 2016/17, 2017/18 and 2018/19 seasons. Influenza B analysis includes 2011/12, 2012/13, 2014/15, 2015/16 and 2017/18 seasons.
